# Identification of Putative Mek1 Substrates during Meiosis in *Saccharomyces cerevisiae* Using Quantitative Phosphoproteomics

**DOI:** 10.1371/journal.pone.0155931

**Published:** 2016-05-23

**Authors:** Raymond T. Suhandynata, Lihong Wan, Huilin Zhou, Nancy M. Hollingsworth

**Affiliations:** 1 Biochemistry and Cell Biology, Stony Brook University, Stony Brook, New York, 11794–5215, United States of America; 2 Ludwig Institute for Cancer Research, University of California San Diego, La Jolla, California, 92093, United States of America; 3 Cellular and Molecular Medicine, University of California San Diego, La Jolla, California, 92093, United States of America; Tulane University Health Sciences Center, UNITED STATES

## Abstract

Meiotic recombination plays a key role in sexual reproduction as it generates crossovers that, in combination with sister chromatid cohesion, physically connect homologous chromosomes, thereby promoting their proper segregation at the first meiotic division. Meiotic recombination is initiated by programmed double strand breaks (DSBs) catalyzed by the evolutionarily conserved, topoisomerase-like protein Spo11. Repair of these DSBs is highly regulated to create crossovers between homologs that are distributed throughout the genome. This repair requires the presence of the mitotic recombinase, Rad51, as well as the strand exchange activity of the meiosis-specific recombinase, Dmc1. A key regulator of meiotic DSB repair in *Saccharomyces cerevisiae* is the meiosis-specific kinase Mek1, which promotes interhomolog strand invasion and is required for the meiotic recombination checkpoint and the crossover/noncrossover decision. Understanding how Mek1 regulates meiotic recombination requires the identification of its substrates. Towards that end, an unbiased phosphoproteomic approach utilizing Stable Isotope Labeling by Amino Acids in Cells (SILAC) was utilized to generate a list of potential Mek1 substrates, as well as proteins containing consensus phosphorylation sites for cyclin-dependent kinase, the checkpoint kinases, Mec1/Tel1, and the polo-like kinase, Cdc5. These experiments represent the first global phosphoproteomic dataset for proteins in meiotic budding yeast.

## Introduction

Meiosis is a specialized type of cell division that produces gametes necessary for sexual reproduction. In meiosis, one round of DNA replication is followed by two rounds of chromosome segregation, Meiosis I (MI) and Meiosis II (MII). The resulting gametes contain half the chromosome number of the parental diploid cell. Proper segregation at MI requires connections between homologous chromosomes created by a combination of sister chromatid cohesion and interhomolog crossovers [1]. Defects in the regulation and execution of meiotic recombination can lead to aneuploidy, which, in human pregnancies, is mostly attributable to errors at MI in mothers [2].

Meiotic recombination is initiated by programmed double strand breaks (DSBs) catalyzed by the meiosis-specific endonuclease, Spo11, usually at discrete regions of the genome known as hotspots [3, 4]. After cleavage, the 5’ ends of the breaks are resected and the 3’ ends are bound by the mitotic recombinase Rad51, as well as the meiosis-specific recombinase Dmc1, to form nucleoprotein filaments that mediate strand invasion of homologous chromosomes [5–8]. DSBs can be repaired either as crossovers (COs) via a pathway that requires a set of functionally diverse proteins called the “ZMM” proteins, or by non-crossovers (NCOs) via synthesis-dependent strand annealing [9–11]. These steps are regulated both directly and indirectly by a meiosis-specific kinase called Mek1/Mre4 [12].

DSB formation triggers the meiotic recombination checkpoint, which is active during normal meiosis, and is spearheaded by Mec1 and Tel1, homologs of the mammalian ATR and ATM kinases, respectively [13, 14]. Mec1/Tel1-phosphorylation of the meiosis-specific axial element protein, Hop1, recruits Mek1 to chromosome axes via its FHA domain where the kinase becomes activated by autophosphorylation *in trans* of threonine 327 [15–17]. Locally activated Mek1 is then able to regulate both the meiotic recombination checkpoint, which monitors the progression of DNA repair, interhomolog bias, and interhomolog recombination pathway choice [18–20].

The purpose of the meiotic recombination checkpoint is to monitor recombination and ensure that meiotic progression does not occur prior to the repair of the DSBs [14]. Deletion of *dmc1Δ* generates resected DSBs that are unable to undergo strand invasion resulting in a checkpoint mediated meiotic prophase arrest [21–23]. Mek1 kinase activity is constitutively required in *dmc1Δ* diploids to maintain this arrest by preventing the repair of DSBs by Rad51 [17]. Rad51 activity requires Rad54 as an accessory factor [24–26]. Mek1 inhibits Rad51 by preventing Rad51-Rad54 complex formation in two independent ways: (1) phosphorylation of Rad54 threonine 132 which decreases the affinity of Rad54 for Rad51 and (2) phosphorylation of Hed1, a meiosis-specific protein that binds to Rad51, thereby excluding Rad54 [27–29](A. Neiman and N. M. Hollingsworth, unpublished data). Inhibition of an analog sensitive version of *MEK1*, *mek1-as*, results in DSB repair using sister chromatids in both *DMC1* and *dmc1Δ* strains [17, 19, 30].

The key to understanding how Mek1 regulates meiotic recombination is the identification of its substrates. Candidate approaches have revealed four *in vivo* substrates, Mek1 T327, Rad54 T132, Hed1 T40, and histone H3 T11 [16, 28, 31] (A. Neiman and N. M. Hollingsworth, unpublished data). To enable unbiased phosphoproteomic screens for proteins phosphorylated by Mek1, a method was developed for sporulating yeast cells after pregrowth in synthetic medium, thereby enabling the application of Stable Isotope Labeling by Amino acids in Cells (SILAC) to meiotic yeast cells [32]. SILAC experiments involve growing cells in medium containing either light or heavy isotope labeled forms of arginine and lysine so that that the proteins derived from the two cultures can be distinguished by their masses. Phosphoproteomics is the approach in which liquid chromatography-tandem mass spectrometry (LC-MS/MS) is used to identify phosphorylation sites on proteins on a proteome wide scale. SILAC has been coupled with phosphoproteomics to identify kinase substrates of ATM/ATR as well as Cdk1 [33, 34].

To identify potential Mek1 substrates, *dmc1Δ mek1-as* diploids were pregrown in either “light” or “heavy” synthetic medium, and then transferred to sporulation medium to initiate meiosis. After arresting the cells in meiotic prophase, Mek1-as was inhibited in the heavy culture for 20 minutes, crude chromatin was isolated from both cultures and combined in equal amounts. The proteins were broken down into peptides by trypsin and phosphopeptides were enriched using immobilized metal affinity chromatography (IMAC) [35]. Proteins phosphorylated by Mek1-as should be under-represented in the heavy culture and therefore the number of light phosphopeptides from Mek1 substrates should be greater than the number of heavy phosphopeptides, thereby giving a light:heavy (L/H) ratio > 2. Manual inspection of the phosphopeptide dataset revealed two known substrates of Mek1, Mek1 T327 and Rad54 T132, thereby validating the approach [32].

This work reports an unbiased bioinformatic analysis of the phosphopeptide dataset generated by the *dmc1Δ mek1-as* SILAC experiments reported in [32]. By looking for kinase consensus motifs enriched in phosphopeptides with L/H ratios >2, a list of potential Mek1 substrates was generated, as well as proteins potentially phosphorylated by the Mec1/Tel1 checkpoint kinases and Cdk1. Because Mek1 activity is required to prevent DSB repair and maintain the checkpoint arrest, inactivation of Mek1 allows meiotic progression. Phosphopeptides with ratios <0.5 may arise from phosphorylation by other kinases in response to Mek1 inactivation. Cdc5 is a polo-like kinase that is induced in meiosis and required for Holliday junction resolution, synaptonemal complex disassembly and spindle pole body duplication [36, 37]. Potential Cdc5 substrates were also revealed by this analysis. In addition to helping understand Mek1 function, this global phosphopeptide dataset may be useful for scientists studying diverse processes in meiosis.

## Results

### The Mek1 consensus motif (RXXT) is enriched specifically in phosphopeptides with L/H ratios >2

**Fig 1:**
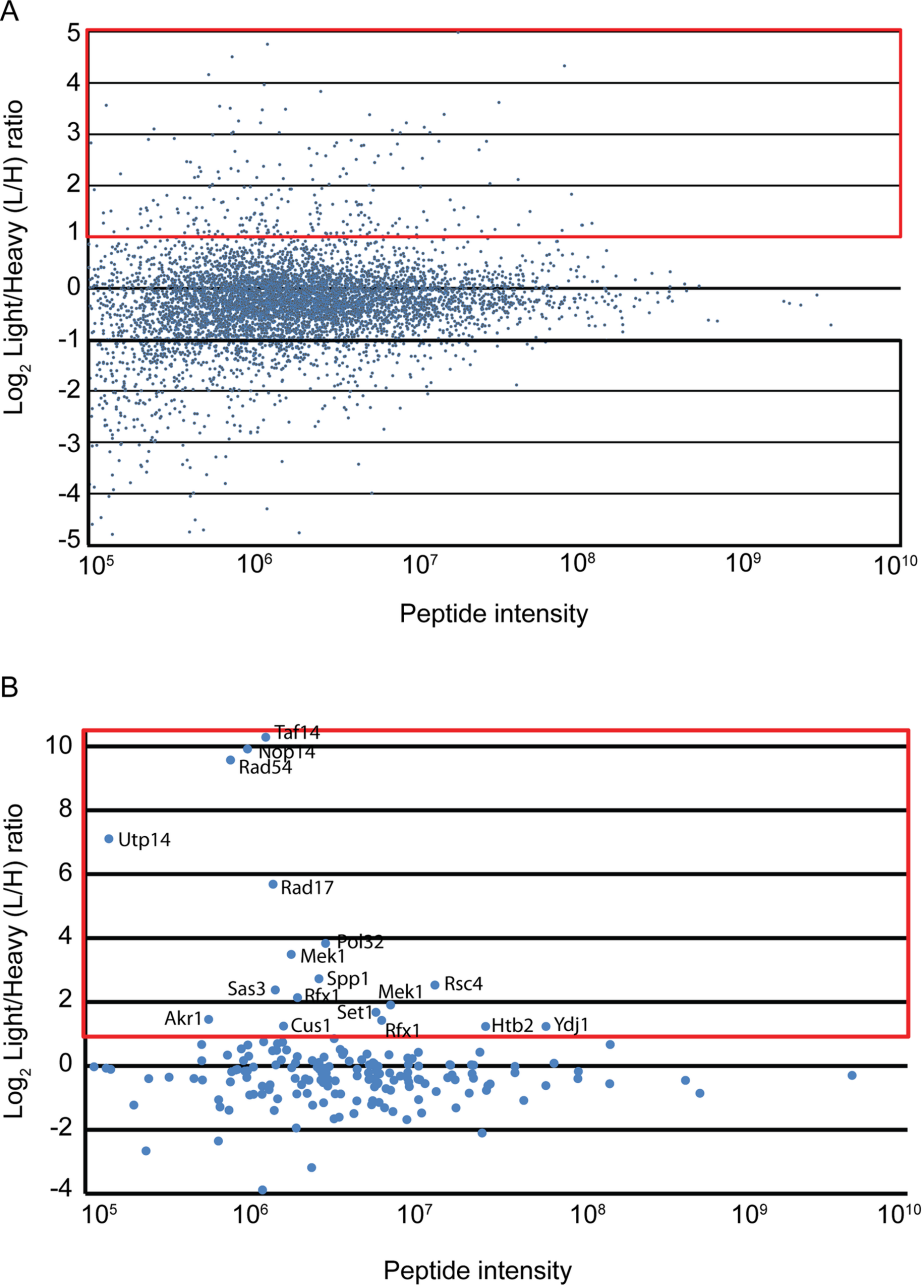
Integrated light/heavy (L/H) ratios of phosphopeptides obtained from the merged non-redundant phosphopeptide datasets from two SILAC experiments using a *dmc1Δ mek1-as* diploid. (A) L/H ratios of phosphorylated peptides are plotted on a log_2_ scale (Y-axis) as a function of peptide intensity (X-axis). The red box indicates Class 1 peptides with L/H ratios > 2.0, while the black box indicates Class 3 peptides with L/H ratios < 0.5. Peptide intensity is a measure of how well each peptide is ionized and subsequently detected by the MS and is correlated to the quantity of each peptide in the sample. Highly abundant peptides will display peptide intensities with large signal to noise ratios and therefore have more accurate L/H ratios when compared to lower abundance peptides with signal to noise ratios closer to 1. L/H ratios were calculated by dividing the integrated peptide intensities of light and heavy peptides. Integrated peptide intensities are defined as the area under the curve of the peptide elution peak. (B) The L/H ratios of 175 RXXT phosphopeptides were plotted on a log_2_ scale (Y-axis) as a function of peptide intensity (X-axis). The red box indicates peptides that are >2-fold enriched in the Light (Mek1 active) culture. The name indicates the protein from which the phosphopeptide was derived.

For analysis, two *dmc1Δ mek1-as* SILAC datasets were merged into one non-redundant set of 6,609 peptides (S1 Table). The peptides were then divided into three classes based on their light/heavy (L/H) ratios (Table 1). Class 1 is comprised of 329 peptides (representing 273 proteins containing 379 phosphosites due to multiple phosphorylation events) with L/H ratios >2 (indicated by the red box in Fig 1A). These phosphosites are candidates for regulation by Mek1 since inhibition of Mek1-as in the heavy culture should lead to underrepresentation of Mek1 phosphopeptides relative to the light culture [32]. Class 2 contains 5,317 peptides with L/H ratios < 2 but > 0.5. Phosphorylation of the peptides in this class was apparently unaffected by the presence of the Mek1-as inhibitor. Class 3 contains 963 phosphorylated peptides (1050 sites) with L/H ratios < 0.5 (indicated by black box) (Table 1)(Fig 1A). This ratio is indicative of phosphorylation occurring in response to Mek1 inactivation, which allows DSB repair and meiotic progression [16, 28, 32].

**Table 1:** Number of *dmc1Δ mek1-as* phosphopeptides containing consensus motifs with different L/H ratios.

Number of peptides = 8563							
	Number of unique phosphopeptides = 6609						
	Class 1: 329 peptides^a^		Class 2: 5317 peptides		Class 3: 963 peptides		
	p-Thr^b^	p-Ser^b^	p-Thr^b^	p-Ser^b^	p-Thr^b^	p-Ser^b^	
Motif	91	250	1101	4371	233	770	Potential Kinases based on [38]
TP	**26**^**c**^	-	**668**	-	**104**	-	CDK, Fus3, Hog1, Pho85
SP	-	**47**	-	**829**	-	**142**	CDK, Hog1, Pho85
SQ	-	**30**	-	224	-	31	Mec1/Tel1 [39]
RXXT	**18**	-	140	-	30	-	Mek1, Gin4, Hsl1, Ksp1, Psk2, Snf1, Yak1
RXXS	-	22	-	**732**	-	**98**	Gin4, Hsl1,Ksp1, Psk2, Rck2, Snf1, Yak1
SXXD	-	26	-	**455**	-	68	?^d^
SXXXL	-	17	-	**528**	-	64	?
SXXE	-	31	-	**568**	-	80	?
SXXSp	-	23	-	**524**	-	100	?
EXS	-	19	-	298	-	**72**	Cdc5?
SA	-	14	-	276	-	**79**	?
DXS	-	19	-	302	-	**69**	Cdc5

^a^The peptides in each class were sorted based on the phosphorylated amino acid. No consensus motifs were observed for phospho-tyrosine-containing peptides and so were not included in the analysis. Phosphopeptides sorted by motif are listed in S1 Table.

^b^Number indicates the total number of phosphopeptides analyzed by the Motif-X algorithm. In some cases the number of phosphopeptides in a column is greater than the number of peptides analyzed because of overlap (eg. SXXD may also be included in SXXXL).

^c^Bold text indicates motifs that are enriched in either Class 1, 2, or 3

^d^? indicates motifs unassociated with a particular kinase

Although an L/H ratio >2 is suggestive of Mek1 regulation, there may be other reasons for this ratio that do not involve the proteins being substrates of Mek1. For example, if a protein that is phosphorylated at the *dmc1Δ* arrest is dephosphorylated or becomes degraded in response to meiotic progression, then an L/H ratio >2 would be obtained due to the loss of the phosphorylated protein in the heavy sample. Therefore further analysis was performed to determine which of the 329 phosphopeptides are most likely to be Mek1 substrates.

Our analysis used the Motif-X algorithm to ask, in an unbiased way, what phosphorylation consensus motifs were enriched in the *dmc1Δ mek1-as* dataset. These motifs were then compared to previously identified consensus sequences to reveal the potential kinases involved. The Motif-X algorithm works by extracting statistically significant motifs from large data sets of naturally occurring phosphorylation sites [40, 41]. It takes the six residues upstream and downstream of a specified phosphorylated amino acid and determines whether the observed number of occurrences for a particular amino acid at one of these positions is statistically enriched over background. The background probability is calculated using the distribution of amino acids surrounding either serine or threonine in the yeast proteome.

**Fig 2:**
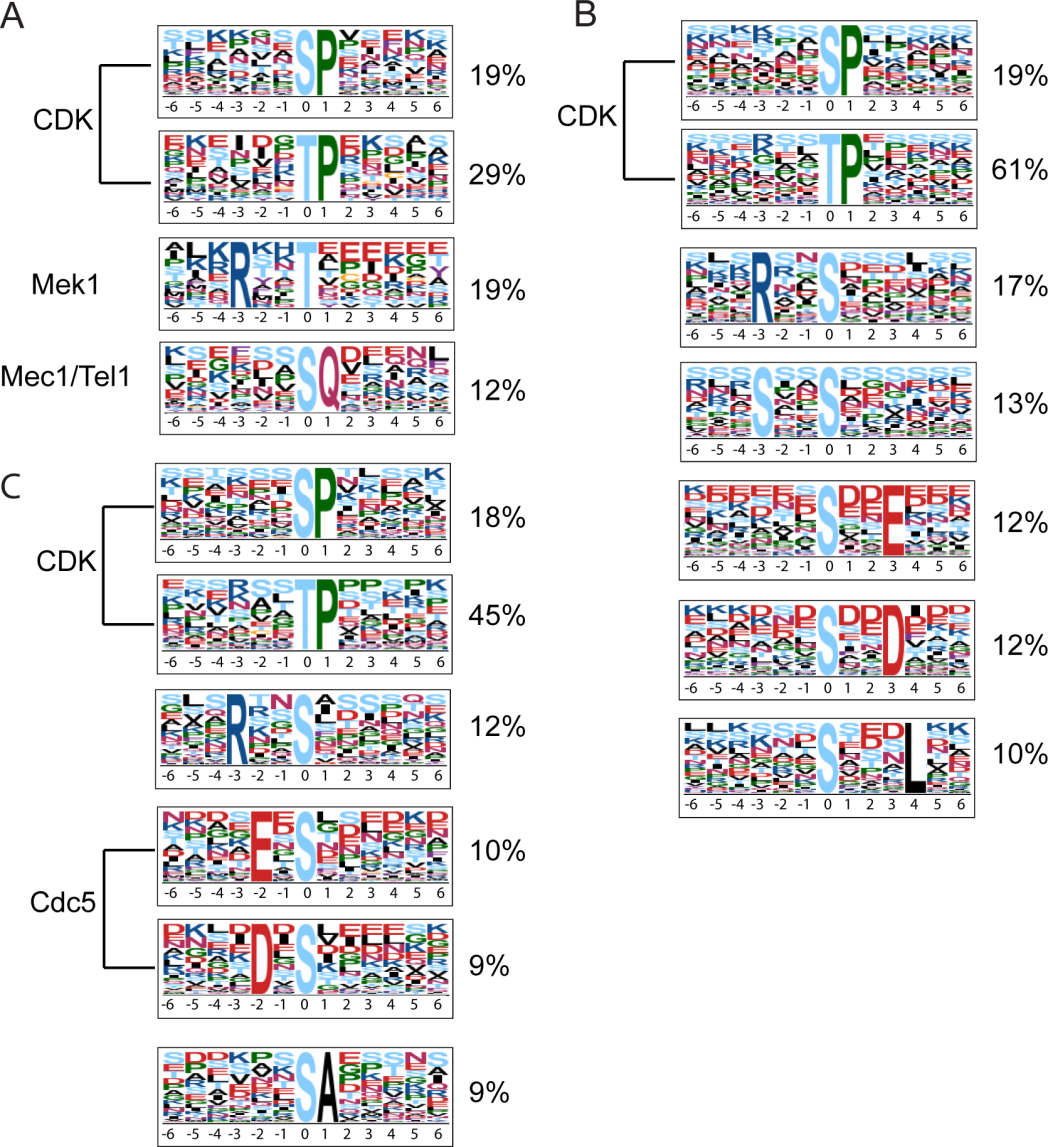
Consensus phosphorylation sites determined by Motif-X analysis of *dmc1Δ mek1-as* SILAC phosphopeptides. (A) Motif-X analysis of the 329 Class I peptides with L/H ratios ≥ 2. (B) Motif-X analysis of the 5317 Class 2 peptides with L/H ratios between 0.5–2.0. (C) Motif-X analysis of the 963 Class 3 peptides with L/H ratios < 0.5. Percentages represent the fraction of peptides containing either phosphothreonine or phosphoserine. The size of each amino acid shows how enriched it is relative to the phosphorylated amino acid, and the color indicates physical and chemical properties of the indicated amino acid (Red: acidic; Dark blue: basic; Black: hydrophobic; Yellow: cysteine; Green: proline/Glycine). The percentages of phosphopeptides containing the motifs are indicated. All of the motifs exhibited significant enrichment with a *p*-value of 0.01.

Motif-X can only analyze one type of phosphorylated amino acid at a time. Therefore enrichment analysis around phosphothreonine must be performed independently from phosphoserine or phosphotyrosine. The 329 Class I phosphopeptides were therefore divided into three query sets: 91 phosphothreonine peptides, 270 phosphoserine peptides, and 13 phosphotyrosine peptides (Table 1). Because no motifs containing phosphotyrosine were detected, perhaps due to the small number of peptides, the phosphotyrosine peptides are not shown in Table 1. To be detected as a motif, the sequence must be present at a frequency of at least 3.96% with a *p*-value of 0.01 (see [41] for a discussion of how the *p*-values are generated). Three consensus sites were detected for the Class 1 peptides: T/SP, SQ, and RXXT (Fig 2A). The T/SP consensus could be indicative of Cdk1 phosphorylation [33], while the SQ motif suggests Mec1/Tel1 phosphorylation [42, 43].

The remaining motif, RXXT, was present in 18 of 96 Class I peptides containing phosphothreonine and is the same as the Mek1 consensus site determined both *in vitro* and *in vivo*. *In vitro*, incubation of peptide libraries with ATP and partially purified GST-Mek1 produced a consensus of RXXT/S [38]. Mek1 phosphorylation sites that occur *in vivo* are all phosphorylated on threonine (Rad54 T132, Mek1 T327, H3 T11 and Hed1 T40) [16, 28, 31] (A. Neiman and N. M. Hollingsworth, unpublished data), consistent with the Motif-X analysis that detected enrichment of RXXT, but not RXXS, in the Class I sites. To test whether enrichment of the RXXT motif is specific to Class 1 as would be predicted for Mek1 targets, Motif-X analysis was performed on the Class 2 and Class 3 phosphosites. Seven motifs were revealed using the 5,317 Class 2 phosphopeptides: TP/SP, RXXS, SXXE, SXXXL, SXXD, and SXXSp (p indicates the phosphorylated residue in cases where multiple serines/theronines are present in the motif) (Fig 2B), while six motifs were identified from the 963 Class 3 phosphopeptides: T/SP, DXS, RXXS, KXXS, SA, SXXSp, EXS, and SSp (Fig 2C). Interestingly the RXXT motif was enriched only in Class 1 peptides, consistent with the phosphates being added by Mek1.

### The Class 1 RXXT candiates putatively regulated by Mek1 contain several proteins involved in DNA metabolism

To determine the distribution of the RXXT motif across the entire dataset, all 175 RXXT sites in the dataset were plotted by their L/H ratios as a function of their peptide intensity. There were more than twice as many phosphosites with L/H ratios > 4 as there were phosphosites with L/H ratios < 0.25, indicating that RXXT sites were enriched in the light culture where Mek1 is still active (Fig 1B). This suggests that RXXT sites that have SILAC ratios that are ≤ 0.25 are likely due to other arginine directed kinases such as Gin4, Hsl1, Ksp1, Psk2, Rck2, Snf1, and Yak1 [38] (Table 1). The 18 Class I RXXT phospho-peptides identify 16 proteins, due to the presence of two RXXT phospho-sites on Mek1 and Rfx1 (Table 2). Two of these sites are confirmed *in vivo* targets of Mek1: Mek1 T327 and Rad54 T132 [16, 28]. Two known substrates were not detected: H3 T11 and Hed1 T40. H3 T11 was likely not observed as the expected tryptic peptide containing the T11 residue is too small to be detected in the mass window used by the MS method. Phosphorylation of Hed1 T40 *in vivo* was originally discovered by LC-MS using whole cell lysates (A. Neiman and N. M. Hollingsworth, unpublished data). In contrast, the *dmc1Δ mek1-as* datasets were generated using crude chromatin. One possible explanation for the failure to detect Hed1 T40 phosphorylation is that Hed1 interacts transiently with chromatin and was lost in the cytosolic fraction during the chromatin enrichment step.

**Table 2:** Class 1 RXXT phosphopeptides.

RXXT protein	Phosphopeptide sequence^a^	Site
Mek1	R.MHT#VVGTPEYCAPEVGFR.A	T327
Mek1	R.AAT#LEQR.G	T356
Rad54	R.SFT#VPIK.G	T132
Spp1	R.NPT#TGEDVYCICK.R	T18
Rad17	R.YGT#DKGNETS#NDNLLQLNGK.K	T350
Rsc4	R.STT#SDIEK.T	T405
Pol32	R.SKT#TPEETTGR.K	T146
Rfx1	R.TNT#FPSIPSSTK.K	T199
Rfx1	R.RNT#QEIIAK.Q	T226
H2B	R.KET#YSSYIYK.V	T39
Taf14	R.RTTT#NTTAEPK.A	T154
Sas3	R.KIT#LIEDDEE.-	T824
Nop14	R.TKT#EEEKNAEAEEK.K	T291
Set7	R.KLT#EEEK.S	T480
Utp14	K.RLDT#YGSGEANEYVLPSANAASGASGK.L	T218
Cus1	R.KHT#AEDELEDT#PSDGIEEHLSAR.K	T104
Akr1	R.YHT#ACQR.G	T79
Ydj1	R.FQT#ECDVCHGTGDIIDPK.D	T183

^a^”.” indicates trypsin cleavage site

“#” follows the phosphorylated amino acid

The fact that known Mek1 substrates were detected raises the possibility that one or more of the remaining 14 proteins is also phosphorylated or regulated by Mek1. To determine which proteins are most likely to be *bona fide* substrates of Mek1, a number of additional criteria were applied. First, since Mek1 is a nuclear protein [44] and all of its known targets are present on chromatin, a reasonable assumption is that additional substrates will also be present in the nucleus. Therefore proteins with cytosolic functions such as Akr1 and Ydj1 are low probability substrates for Mek1 [45–50]. A second assumption is that Mek1 substrates will be associated with DNA processes, making proteins involved in splicing and ribosome assembly/function such as Cus1, Utp14, Set7 and Nop14 low probability substrates as well [51–55]. The remaining proteins all play some role in chromosome metabolism and therefore are more likely to be bona fide Mek1 substrates. H2B is a histone component of nucleosomes, as is histone H3, a known target of Mek1 [31, 56]. Other potential connections between Mek1 and chromatin are Sas3, the catalytic subunit of the NuA3 histone acetyl transferase (HAT) complex that acetylates H3K9 and H3K14 and Rsc4, a subunit of the essential RSC multi-subunit chromatin remodeling complex [57–59]. Mek1 could also be involved in regulating transcription as Taf14 physically associates with the general transcription factors TFIID and TFIIF, the chromatin remodeling complexes SWI/SNF, Ino80 and RSC, and the histone modification enzyme NuA3 [60] and Rfx1/Crt1 is a transcriptional repressor responsible for repressing the DNA damage inducible ribonucleotide reductase (*RNR*) genes [61]. Pol32 is the non-essential subunit of Polδ and is required for the initiation of Rad51-dependent break induced replication (BIR) [62].

With regard to meiotic recombination, two intriguing candidates are Spp1 and Rad17. Spp1 is a subunit of the Set1/COMPASS (COMplex of Proteins ASsociated with Set1) complex [63]. This complex is composed of seven subunits in addition to Set1, which are Bre2, Swd1, Spp1, Swd2, Swd3, Sdc1, and Shg1. In vegetative cells, Set1/COMPASS catalyzes H3K4 methylation, which then recruits chromatin remodelers and promotes gene activation [64–66]. Spp1 has recently been shown to be a bridge that brings hotspot sequences in the loop regions of meiotic chromosomes to the chromosome axes where Spo11 then catalyzes DSBs [67–69]. While deletion of *SPP1* has no effect on spore viability, *spp1**Δ* mutants alter the pattern of DSB formation, with breaks at some hotspots becoming reduced and novel hotspots arising in previously cold regions of meiotic chromosomes [67, 69]. Spp1 T18 phosphorylation has not been previously reported according to PhosphoGRID (http://www.phosphogrid.org/), and therefore could be meiosis specific. Given that Mek1 is activated on the axes where Spp1 is localized, an intriguing possibility is that Mek1 phosphorylation of Spp1 may trigger release of the DSB ends thereby promoting repair.

Rad17 is part of the “9-1-1” complex (Ddc1-Rad17-Mec3) in budding yeast which is involved in the mitotic DNA damage response, as well as the meiotic recombination checkpoint [23, 70]. The 9-1-1 complex has a ring-like structure and is a clamp loaded onto the ssDNA-dsDNA junction by the Rad24-replication factor complex (RFC) [70, 71]. After being loaded, the clamp has the ability to slide across double stranded DNA much like the DNA replication clamp PCNA and is able to promote downstream events such as the activation of Mec1 [70, 72]. *RAD17* is required for efficient meiotic DSB repair and the meiotic recombination checkpoint [23, 73]. In addition, Rad17 is implicated in one of two pathways for Mek1 activation, Mec1 via Rad17 and Tel1 via Pch2 [74]. Deletion of *RAD17* lowers spore viability to 37.1% due to a decrease in interhomolog recombination [73, 74]. A recent study showed that the 9-1-1 complex promotes the assembly of the ZMM proteins at DSBs [75]. *MEK1* promotes phosphorylation of the C terminus of Zip1, one of the ZMM proteins. In the absence of phosphorylation, the Zip1 protein forms foci that have been proposed to be Zip1 bound to DSBs [18]. One possible model is that phosphorylation of Rad17 by Mek1 helps recruit Zip1 to DSBs thereby promoting COs through the ZMM pathway. Alternatively or in addition, phosphorylation of Rad17 by Mek1 could be necessary to maintain the meiotic recombination checkpoint. The T350 phosphorylation site identified on Rad17 has not been previously reported according to PhosphoGRID, and therefore could potentially be meiosis specific.

To test whether the Rad17 T350 phosphorylation is functional *in vivo*, T350 was mutated to alanine (to prevent phosphorylation) or aspartic acid (to act as a phosphomimic). A modest reduction in spore viability was observed for both the *rad17-T350A* and *T350D* mutants, but the differences were not significant (unpaired T-test with Welch correction, P = 0.189) (Table 3). Deletion of *RAD17* has been observed to exacerbate spore inviability when combined with mutants of different genes, such as *PCH2*, a AAA+ ATPase involved, among other things, in regulating the levels of Hop1 on chromosomes, *NDJ1*, a gene required to tether telomeres to the nuclear envelope to promote homolog pairing, and *ZIP1*, which encodes a transverse filament protein of the synaptonemal complex [76–78]. Plasmids containing either *RAD17*, *rad17-T350A* or *rad17-T350D* were introduced into the appropriate double mutants and assayed for sporulation and spore viability. Both *rad17-T350A* and *rad17-T350D* complemented the sporulation and spore viability defects of *rad17Δ* in the *pch2Δ* and *zip1Δ* backgrounds (Table 3). A small reduction in sporulation and spore viability was observed for *zip1-T350A* in the *ndj1Δ* background, but it is not clear whether this difference is significant. These results indicate that phosphorylation of Rad17 is not required for the primary function of *RAD17* during meiosis. This is reminiscent of Mek1 phosphorylation of Rad54 T132: the *RAD54-T132A* mutant fully complements the sporulation and spore viability defects of *rad54Δ* because phosphorylation negatively regulates Rad54 activity and *RAD54* is not required for interhomolog recombination [28, 79]. Nonethless, a robust phenotype was observed in the *dmc1**Δ* background, where the *RAD54-T132A* allowed the repair of DSBs by Rad51. Therefore more work is required to determine the functional significance of Rad17 T350 phosphorylation.

**Table 3:** Sporulation and spore viability in various *rad17* phosphorylation site mutants^a^.

Strain	relevant genotype	% sporulation ± SD	% spore viab. ± SD
NH2341/pRS424	*rad17Δ*	76.0 ± 2.4	15.8 ± 2.6
NH2341::pMR1	*RAD17*	95.7 ± 0.7	94.8 ± 2.2
NH2341::pMR1-T350A	*rad17-T350A*	86.8 ± 13.2	89.6 ± 6.8
NH2341::pMR1-T350D	*rad17-T350D*	90.9 ± 2.4	89.6 ± 6.1
NH2373/pRS316	*pch2Δ rad17Δ*	58.2 ± 12.4	1.0 ± 1.7
NH2373::pLW102	*pch2Δ*	95.5 ± 2.0	90.4 ± 7.2
NH2373::pLW103	*pch2Δ rad17-T350A*	94.9 ± 2.8	87.5 ± 8.2
NH2373::pLW104	*pch2Δ rad17-T350D*	94.0 ± 2.3	89.8 ± 4.8
NH2374/pRS316	*ndj1**Δ* *rad17Δ*	40.6 ± 10.5	4.6 ± 5.5
NH2374::pLW102	*ndj1Δ*	77.6 ± 5.7	55.8 ± 6.8
NH2374::pLW103	*ndj1Δ rad17-T350A*	67.0 ± 13.0	49.2 ± 11.7
NH2374::pLW104	*ndj1Δ rad17-T350D*	76.7 ± 11.8	49.8 ± 4.4
NH2375/pRS316	*zip1Δ rad17Δ*	53.9 ± 20.2	2.1 ± 1.6
NH2375::pLW102	*zip1Δ*	89.8 ± 2.7	45.8 ± 8.1
NH2375::pLW103	*zip1Δ rad17-T350A*	89.9 ± 3.6	49.0 ± 9.4
NH2375::pLW104	*zip1Δ rad17-T350D*	88.9 ± 7.3	46.0 ± 5.8
NH2376/pRS316	*dmc1Δ rad17Δ*	ND	4.6 ± 2.3
NH2376::pLW102	*dmc1Δ*	ND	0.0 ± 0.0
NH2376::pLW103	*dmc1Δ rad17-T350A*	ND	0.0 ± 0.0
NH2376::pLW104	*dmc1Δ rad17-T350D*	ND	0.3 ± 0.4

^a^Raw data are presented in S2 Table.

### Class 1 proteins identify putative substrates of the Mec1/Tel checkpoint kinases

The Class 1 sites with SQ motifs may identify Mec1/Tel1 substrates important for the activation and maintenance of the meiotic recombination checkpoint. Because inhibition of Mek1 results in repair of DSBs using sister chromatids, the checkpoint is no longer activated, thereby allowing the removal of Mec1/Tel1-mediated phosphorylation. These sites should therefore be under-represented in the heavy culture, resulting in L/H ratios >2. There were 29 unique SQ sites, representing 28 proteins, present in the Class 1 phosphopeptide dataset (S1 Table). Further classification of the SQ proteins was performed based on their biological functions. The criteria used to separate the SQ proteins was: 1) Proteins that are involved in the mitotic and/or meiotic recombination checkpoint 2) Proteins that exhibit sensitivity to DNA damage when their genes are mutated and 3) proteins previously identified as Mec1/Tel1 targets. Nine proteins, Red1, Mec1, Zip1, Ies4, Rtt107, Ioc2, Rfa2, Spt7, and Rfx1 all satisfy at least one of the three criteria mentioned above, while Mec1, Ies4, Rtt107, Ioc2 and Rfa2 are sensitive to DNA damage and are previously identified Mec1/Tel substrates [80, 81]. Zip1 encodes the meiosis-specific transverse filament protein of the SC and is required for non-homologous centromere coupling and the ZMM pathway responsible for generating crossovers distributed by interference and synapsis [10, 82–84]. Zip1 has been previously been found to be phosphorylated by Mec1 on S75, which regulates non-homologous centromere pairing early in meiotic prophase [85]. Red1 is a meiosis-specific component of axial elements that physically interacts with Hop1 and is required for Mek1 activation [16, 86, 87]. Red1 is a phosphoprotein that exhibits a mobility shift on sodium-dodecyl-sulfate polycracrylamide gels [86, 88]. However, this shift is independent of Mec1/Tel1, Cdk and Mek1 [89]. Therefore if the putative Mec1 site is functionally important, phosphorylation of S597 must not contribute to this shift. Phenotypic analysis of mutants in these putative Mec1/Tel1 phosphosites could determine whether phosphorylation of these proteins is important for activation or maintenance of the meiotic recombination checkpoint.

### Class 3 phosphosites may reveal substrates of the polo-like kinase Cdc5

One way that the low L/H ratios observed for Class 3 phosphosites could occur is if the proteins were phosphorylated in response to Mek1 inactivation. Because inactivation of *mek1-as* in the *dmc1**Δ* background results in DSB repair using sister chromatids, the cells in the heavy culture were no longer arrested by the meiotic recombination checkpoint and were able to progress through meiosis [32]. Meiotic progression requires induction of *NDT80*, a meiosis-specific transcription factor that is responsible for the expression of >200 genes, including the gene including the polo-like kinase, *CDC5*, and the cyclin-dependent kinase, *CLB1* [90, 91]. Therefore substrates of both Cdc5 and Cdk1 would be predicted to occur in the heavy but not the light culture in the *dmc1Δ mek1-as* SILAC experiment. In fact, phosphopeptides containing the Cdk consensus T/SP are enriched in Class 3, as well as the DXS motif, which closely matches the D/EXS/Tψ (where ψ represents a hydrophobic residue) consensus of Polo-like Kinase (PLK) [92](Fig 2)(S1 Table). Furthermore, Polo-like Kinase is known to have conserved Polo-box domains that target the kinase to its substrates [93]. The human homolog of Cdc5, Plk1, preferentially binds phosphopeptides sequences of Ser-pThr/pSer-Pro/X [94]. *CDC5* is required for many cell division processes, including Cdk1 activation, spindle formation, cohesin removal from chromosome arms, and cytokinesis [95]. In budding yeast, Cdc5 has been shown to play essential roles in mitotic exit and cytokinesis [93]. In meiotic cells, ectopic expression of *CDC5* at the *ndt80Δ* arrest is sufficient for JM resolution and SC disassembly [37]. Cdc5 is also necessary for cleavage and removal of cohesion from chromosome arms and mono-orientation of sister kinetochores during meiosis I [96].

Candidate Cdc5 substrates meet the following criteria: 1) Phosphopeptides with L/H ratio < 0.5; (2) contain the D/EXS/Tψ Plk consensus motif and (3) are on the same protein containing a Ser-pThr/pSer-Pro/X Polo-box binding motif. Out of the 963 class 3 phosphopeptides, 13 sites (representing 10 proteins) contained the Plk phosphorylation motif of D/EXS/Tψ consensus motif and the Polo-box binding domain of Ser-pThr/pSer-Pro/X (Table 4). A potentially interesting substrate is Zip3, which is a SUMO ligase and one of the ZMM proteins involved in the formation of double Holliday junction intermediates that exhibit biased resolution to give crossovers [10, 97]. Holliday junction resolution requires Cdc5, so it is tempting to speculate that phosphorylation of Zip3 by Cdc5 regulates resolution [37].

**Table 4:** Class 3 proteins containing D/EXS/Tψ and Polo-box binding motifs.

Protein	Peptide^a^	Site
Abp1	DSEFNSFLGTTKPPSMTESS#LK	S296
Cst9/Zip3	SSDIS#IINLVESK	S97
Isw1	IREEFADQT#ANEKENVDGVESK	T1089
Lsb3	SFAGEES#GDLPFR	S408
Net1	DIS#LHSpLK	S744
Nop4	ITGQNNEDEDDADGEDS#MLK	S141
Nup60	ISSMPGGYFHSEIS#PDSTVNR	S81
Nup60	SAEGNNIDQS#LILK	S171
Nup60	SNVVVAETS#PEKK	S382
Rif1	LEDS#GTCELNK	S1694
Rif1	DIS#VLPEIR	S1755
Sef1	LNLHPTPTPGTIIPNPDSS#PSSpGSPTSSAAQR	S160
Shp1	NTFAGGETS#GLEVTDPSDPNSLLK	S155

^a^ Phosphorylated residues containing the D/EXS/Tψ motif are followed by “#” while other phosphorylated residues are indicated by a “p” after the phosphorylated residue.

## Discussion

Quantitative proteomics has been widely used to elucidate many biological questions over the last decade [98]. SILAC, coupled with MS, has been a simple and powerful tool to improve the quality of data generated from quantitative high throughput proteomic studies [99]. SILAC, unlike post-digestion labeling techniques such as isotope-coded affinity tags, uses cells’ natural metabolic pathways to strategically label the proteome with stable heavy isotope labeled amino acids [100]. The nature of this labeling procedure allows for consistent labeling efficiency of a cell’s proteome, and has been extensively used in the last decade to obtain robust high-throughput data in various proteomic studies [101–103]. SILAC is versatile and has been successfully applied to a variety of model organisms including yeast, fruit flies, plants and mice [80, 104–107].

The recent development of a protocol for sporulating yeast cells out of synthetic medium has allowed the application of SILAC for the identification of phosphorylation sites in meiosis for the first time [32]. A SILAC experiment using *mek1-as* cells arrested in meiotic prophase by *ndt80Δ* revealed that the C-terminus of Zip1 is phosphorylated in a Mek1-dependent manner [18]. This work reports the first global dataset of proteins phosphorylated during budding yeast meiosis. This dataset includes many meiosis-specific proteins that were lacking from previous phosphoproteomic analyses that used vegetative cells. The primary goal was to look for Mek1 substrates. The *dmc1Δ* mutant was used to arrest cells with unrepaired DSBs. This arrest requires constitutive Mek1 activity, both to prevent repair of the breaks by Rad51 and to maintain the meiotic recombination checkpoint [20, 30]. Because inactivation of Mek1-as in the heavy culture allows repair and meiotic progression, the SILAC ratios not only reveal potential targets of Mek1 (which have L/H ratios >2), they may also indicate proteins that become phosphorylated in response to Mek1 inactivation (L/H <0.5). Manual inspection of this dataset previously detected Rec8, the meiosis-specific cohesion subunit, as one such protein [108]. Many of the Rec8 phosphosites detected with ratios < 0.5 were previously identified and shown to be important for cohesin cleavage at anaphase [32, 109, 110].

Over 6000 phosphopeptides were analyzed in an unbiased way for the presence of enriched sequence motifs. The fact that the Mek1 consensus, RXXT, was enriched only in the Class 1 peptides with L/H ratios >2 suggests that these proteins may be bona fide targets of Mek1. Preliminary experiments mutating a putative Mek1-dependent phosphosite on Rad17 indicate that phosphorylation at this site is not required for *RAD17* function in meiosis, but may instead either be redundant with other phosphosites or regulatory in nature. Nevertheless, the list provides a starting point for thinking about the roles that Mek1 may play in meiotic chromatin, replication and transcription. In addition, our analysis has generated lists of potential Mec1/Tel1, Cdk and Cdc5 targets, as well as a global dataset of meiosis-specific phosphoproteins.

## Materials and Methods

### Yeast strains

All strains were derived from the SK1 background. Genotypes are shown in Table 5. Sporulation was carried out at 30°C as described in [111]. Genes were deleted by polymerase chain reaction (PCR)-based methods using *kanMX6*, *natMX4*, *hphMX4*, markers that confer resistance to G418, nourseothricin and Hygromycin B, respectively [112, 113]. All deletions were checked by PCR to confirm both the presence of the deletion and the absence of the wild-type allele. The different *RAD17* alleles were carried on plasmids that were targeted to integrate upstream of *rad17Δ*::*natMX4* by digestion with MluI (see below).

**Table 5:** *S*. *cerevisiae* strains.

Strain	Genotype	Source
NH2092	*MATα leu2/MAT***a***leu2 arg4-Nsp/* *arg4-Nsp* *hoΔ*::*LYS2/* *hoΔ*::*LYS2* *lys2/* *lys2* *ura3/* *ura3* *lys4Δ*::*hphMX4/l**ys4Δ*::*hphMX4* *dmc1Δ*::*LEU2/* *dmc1**Δ*::*LEU2* *mek1Δ*::*kanMX6*::*URA3*::*mek1-as/* *mek1Δ*::*kanMX6*::*URA3*::*mek1-as*	[32]
NH716	*MATα/* *MAT****a*** *leu2*::*hisG his4-X*::*LEU2 (NgoMIV)/* *leu2*::*hisG HIS4*::*LEU2* *hoΔ*::*hisG/)* *ho**Δ*::*hisG* *ura3(Δpst-sma**)/ura3(Δpst-sma)*	[114]
NH2341	same as NH716 only *trp1-5’**Δ*::*hphMX4 rad17**Δ*::*natMX4*	this work
NH2373	same as NH716 only *pch2**Δ*::*kanMX6 rad17**Δ*::*natMX4*	this work
NH2374	same as NH716 only *ndj1**Δ*::*hphMX4 rad17**Δ*::*natMX4*	this work
NH2375	same as NH2341 only *zip1**Δ*::*kanMX6*	this work

### Plasmids

To construct a *RAD17 TRP1* integrating plasmid, a 1.7 kb fragment containing the *RAD17* open reading frame and flanking sequences was synthesized with BamHI and SalI ends and cloned into pUC57 by Genewiz. This fragment was subsequently subcloned into BamHI/SalI-digested pRS304 [115] to generate pMR1. The T350 codon was mutated either to GCA for alanine or GAT or aspartic acid by site-directed mutagenesis (QuikChange II Site-Directed Mutagenesis kit from Agilent Technologies). The mutant alleles were sequenced in their entirety by the Stony Brook University DNA Sequencing facility to confirm the presence of the mutations and the absence of any unintentional mutations. The 1.7 kb BamHI/Sal1 fragments were subsequently subcloned from pMR1 (*RAD17*), pMR1-T350A (*RAD17-T350A*) or pMR1-T350D (*RAD17-T350D*) into BamHI/SalI digested pRS306 (a *URA3* integrating vector) [115] to make pLW102, pLW103 and pLW104, respectively.

### SILAC experiments

A detailed description of the experiments used to generate the phosphopeptide dataset analyzed in this paper is presented in [32].

### Motif X analysis

The Motif-X analysis was performed using an algorithm which can be accessed using the website, http://motif-x.med.harvard.edu/motif-x.html [40, 41].

## Supporting Information

S1 Table Phosphopeptides obtained from two *dmc1**Δ* *mek1-as* SILAC experiments. The non-redundant phosphopeptides are listed alphabetically by gene name. Phosphorylation is indicated by “+80” immediately following the modified amino acid in the peptide sequence. The amino acid position within the protein is indicated in brackets after the open reading frame (ORF) name. “L area” and “H area” indicate the integrated peptide intensities defined as the area under the curve of the peptide elution peak. “L/H” is the ratio of the L and H areas.
(XLSX)

S2 Table Sporulation and spore viability in various *rad17Δ* diploids. The number of viable spores in each tetrad are presented for each patch dissected as well as the average spore viability and standard deviation (SD) between the patches. Sporulation was assayed by light microscopy. Two hundred cells were counted for each patch.
(XLSX)
